# Large area laser scanning optical resolution photoacoustic microscopy using a fibre optic sensor

**DOI:** 10.1364/BOE.9.000650

**Published:** 2018-01-18

**Authors:** Thomas J. Allen, Olumide Ogunlade, Edward Zhang, Paul C. Beard

**Affiliations:** Department of Medical Physics and Biomedical Engineering, University College London, Gower Street, WC1E6BT, UK

**Keywords:** (110.5120) Photoacoustic imaging, (170.3880) Medical and biological imaging

## Abstract

A laser scanning optical resolution photoacoustic microscopy (LS OR-PAM) system based on a stationary fibre optic sensor is described. The sensor comprises an optically resonant interferometric polymer cavity formed on the tip of a rounded single mode optical fibre. It provides low noise equivalent pressure (NEP = 68.7 Pa over a 20 MHz measurement bandwidth), a broad bandwidth that extends to 80 MHz and a near omnidirectional response. The latter is a significant advantage, as it allows large areas (>1cm^2^) to be imaged without the need for translational mechanical scanning offering the potential for fast image acquisition. The system provides a lateral resolution of 8 µm, an axial resolution of 21 µm, and a field of view up to 10 mm × 10 mm. To demonstrate the system, *in vivo* 3D structural images of the microvasculature of a mouse ear were obtained, showing single capillaries overlaying larger vessels as well as functional images revealing blood oxygen saturation.

## 1. Introduction

Optical Resolution Photoacoustic Microscopy (OR-PAM) can provide high resolution micron scale images of the superficial (<1 mm) microvasculature and other absorbing structures [1]. It relies on raster scanning a focused excitation laser beam over the surface of the tissue sample and detecting the generated photoacoustic signals using an ultrasound detector. The lateral resolution of the image is defined by the size of the focal spot, and the axial resolution is defined by the bandwidth of the detected photoacoustic signal. The latter depends on the bandwidth of the ultrasound detector and the effects of frequency-dependent acoustic attenuation of the tissue sample. The depth penetration is defined by the ability to focus light within biological tissue and is typically limited to less than 1 mm due to optical scattering.

Early implementations [2,3] relied on mechanically scanning an assembly composed of a microscope objective and an ultrasound detector over the tissue sample, or translating the tissue sample while keeping the assembly stationary. However, the inertial limitations of translational mechanical scanning limits acquisition speed. Faster acquisition can be achieved using water-immersible microelectromechanical based systems (MEMS). These comprise a submersible scanning mirror that delivers the light to the sample and the returning acoustic wave back to a stationary ultrasound detector [4,5]. Two configurations have been developed. In the first configuration, a 1-axis MEMS mirror is used to fast scan along the x-axis and slower translational mechanical scanning is used to scan along the y-axis, limiting the maximum imaging speed. In the second configuration, a 2-axis MEMS mirror is used, removing the need for any translational mechanical scanning. However, the reported scanning speeds of 2-axis MEMS are much slower (B-scan rate of 50 Hz over a 3 mm range) [5] than that those achieved with 1-axis MEMS (B-scan rate of 400 Hz over a 3 mm range) [4]. An alternative approach is Laser Scanning Optical Resolution Photoacoustic Microscopy (LS OR-PAM) [6–10]. This method uses an x-y galvanometer scanner to optically scan the focused excitation beam, while detecting the generated photoacoustic signals with a single stationary ultrasound detector. The absence of translational mechanical scanning allows potentially faster acquisition speeds to be achieved, limited only by the speed of the galvanometers (B-scan rate of 2000 Hz over a 5 mm range) and the pulse repetition frequency (PRF) of the excitation laser. LS OR-PAM has been implemented using focused [11,12] and planar [6,9] piezoelectric detectors, as well as optical detectors [7,13]. Focused piezoelectric detectors provide high sensitivity, but at the cost of a small field of view (FOV) (<∅1 mm) which is defined by the focal spot size of the ultrasound detector. Planar piezoelectric detectors allow for larger FOVs to be imaged, defined by the element size of the detector and its acceptance angle. However, to date, the largest area imaged *in-vivo* using LS OR-PAM was 3.5 mm x 3.5 mm using a 6 mm diameter piezoelectric planar detector [6]. The use of such a large element size with its inevitably small acceptance angle requires the detector to be placed a significant distance (3 cm) from the sample resulting in increased acoustic attenuation and a relatively bulky setup. Using a small element size detector (<100 µm) with a wide acceptance angle can overcome these limitations, as the sensor can be placed close to the sample whilst still providing a large field of view. However, it is challenging to manufacture small piezoelectric detectors with element sizes on the tens of micron scale with sufficiently high sensitivity as their sensitivity scales with area. Optical sensors such as micro ring resonators (MRR) [7] can provide small element size sensors with acceptance angles larger than those achieved using piezoelectric sensors and with low NEP; however, the ability to image large areas (>2 mm) has not previously been demonstrated.

In this study, we seek to exploit the high acceptance angle of a fibre optic ultrasound sensor based on an optically resonant interferometric polymer cavity to image larger FOVs than previously reported LS OR-PAM implementations. Previous preliminary studies [13,14] have demonstrated the concept by acquiring a single wavelength 2D image using a 1st generation sensor. In this paper, we describe the use of a different sensor with improved frequency response and directional characteristics, a detailed description of the scanner hardware and imaging performance and the use of a tunable laser system to acquire multi-wavelength images. Section 2 describes the experimental setup. Section 3 characterises the LS OR-PAM system and the fibre optic sensor. Section 4 discusses the *in-vivo* experiments, demonstrating that the system can provide high resolution 3D images of the microvasculature over a large FOV.

## 2. Experimental setup

**Fig. 1:**
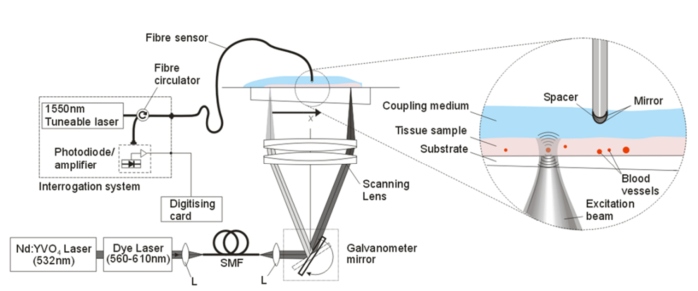
Experimental setup. (SMF: Single Mode Fibre, L: Lens).

The experimental setup is shown in Fig. 1

. A dye laser, pumped by a frequency doubled, Q-switched Nd:YVO_4_ laser (Elforlight, UK), was used to generate the photoacoustic signals. The laser provided pulse energies up to 5 µJ, pulse durations of 1.2 ns and was tunable across the 560 nm to 610 nm wavelength range. The direct output of the pump laser (532 nm) was also accessible and used for some of the experiments. The output of the laser was coupled into a single mode fibre (P3-460B-FC-2, Thorlabs ltd) to spatially filter the beam, and the divergent beam exiting the fibre was then collimated (F220APC-532 Thorlabs Ltd). A pair of galvanometers (Cambridge Technology Ltd.) was used to raster scan the collimated beam via a scanning lens. The scanning lens was composed of a pair of achromatic lenses (AC508-100-A, Thorlabs Ltd) that provided a focal length of 5 cm, and provided a focal spot size of 8 µm (FWHM). The pair of galvanometers was controlled using a multifunction data acquisition card (PCI-6323 National Instruments) via a Labview program. The focused excitation beam was scanned over the tissue sample, which was positioned on a Perspex substrate placed above the pair of achromatic lenses. Ultrasound gel was used as an acoustic coupling agent between the tissue sample and the fibre optic sensor.

The fibre optic sensor comprises an optically resonant polymer cavity formed at the tip of an optical fibre [14]. The transduction mechanism is one in which an incident acoustic wave modulates the optical thickness of the cavity and thus its reflectivity which is read-out using a cw laser beam coupled into the core of the fibre. The sensor was fabricated by depositing a dielectric mirror coating with ~98% reflectivity on the rounded tip of a single mode fibre with a 10 µm core diameter, followed by a dome shaped epoxy spacer of 11.8 µm thickness and a second dielectric mirror. A 10 µm thick Parylene-C barrier coating was applied to protect the sensor. By using a rounded-tip single mode fibre, as opposed to a plane-cleaved one [14], acoustic diffraction can be reduced [15], enabling superior frequency response and directional characteristics to be achieved. The sensor was interrogated using a continuous wave tunable laser (Tunics T100S-HP CL, Yenista Optics, France) operating in the 1500-1600 nm wavelength range. An optical circulator was used to deliver the light from the tunable laser source to the fibre optic sensor and the reflected light to a fibre coupled InGas photodiode-transimpedance amplifier unit with a 100 MHz bandwidth. The amplifier provided separate AC and DC coupled outputs. In order to record the photoacoustic signal, the AC output was connected to a fast 8-bit digitising card (PCI-5114 National Instruments) with a 250 MS/s sampling rate. The DC output was connected to the multifunction data acquisition card (250 kS∕s 16-bit analogue-to-digital (A/D) card PCI-6323 National Instruments) and allowed for the transfer function of the sensor to be recorded in order to determine the optimum bias of the sensor. The biasing of the sensor involves tuning the wavelength of the interrogation laser so that it corresponds to the peak derivative of the cavity transfer function [16] where the sensitivity and linearity of the sensor is maximised.

## 3. System characterisation

**Fig. 2:**
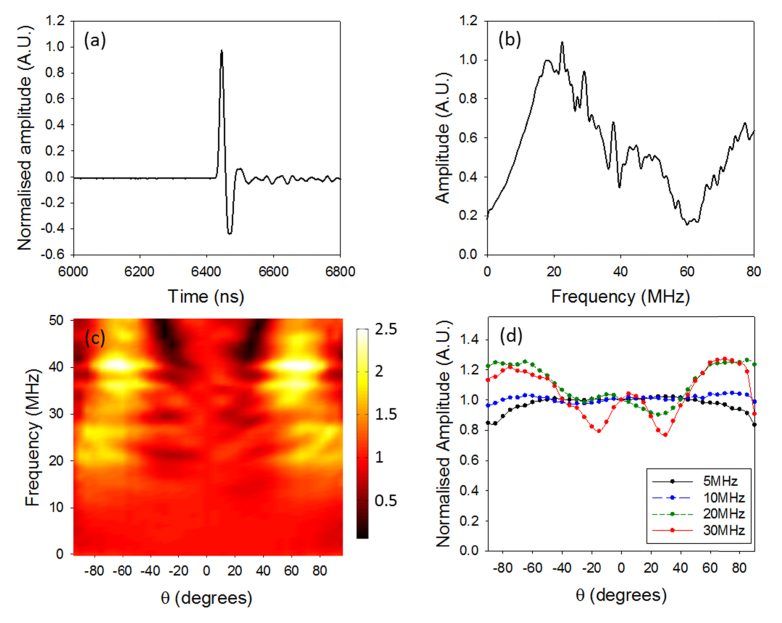
Acoustic characteristics of the optical fibre sensor, (a) Impulse response, (b) Frequency response, (c) Directional response (normalised to θ = 0°), (d) profile through (c) for selected frequencies [15].

To determine the frequency response of the sensor, its impulse response was measured (Fig. 2(a)

). The measurement was acquired by detecting a normally incident broadband (100 MHz) monopolar acoustic plane-wave generated by illuminating a thin planar black absorber with a 5 ns laser pulse emitted by a Q-switched Nd:YAG laser. The frequency response of the fibre optic sensor was then obtained by dividing the frequency spectrum of the measured impulse by that of the source, which was measured independently using a calibrated 130 MHz planar FP sensor of known frequency response [15]. Figure 2(b) shows the frequency response of the fibre optic sensor illustrating that its detectable frequency range extends to 80 MHz. The directivity of the sensor was also measured by varying the incidence angle of the monopolar plane-wave [15,17]. Figures 2(c) and 2(d) show that the sensor provides a near omnidirectional response at frequencies as high as 30 MHz. This allows the sensor to be placed in close proximity (a few mm) to the sample in order to reduce acoustic attenuation while still being able to achieve a large FOV (>1 cm^2^). The noise-equivalent pressure (NEP) was also determined by comparing the output of the fibre optic sensor to that of a calibrated 130 MHz (−3 dB bandwidth) planar FP sensor of known sensitivity [15]. NEP was defined as the acoustic pressure that provides a signal-to-noise of unity. The noise was estimated, over a 20 MHz measurement bandwidth without signal averaging, by calculating the root-mean-square value from a segment taken from immediately before the arrival of the acoustic pulse. The NEP was estimated at 9 MHz to be 68.7 Pa over a 20 MHz bandwidth. Compared to the plane-cleaved fibre sensor reported in reference [14], these results show that the rounded-tip sensor provides almost a factor of two higher bandwidth, smoother frequency response and improved directivity but higher NEP.

**Fig. 3:**
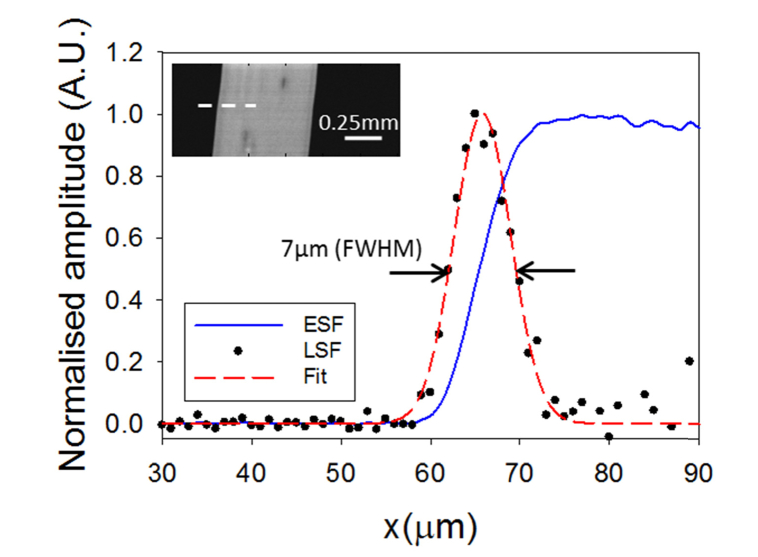
Edge spread function (ESF) obtained from the sum of profiles obtained through the photoacoustic image of a black absorbing ribbon (see inset) and line spread function (LSF) calculated by taking the derivative of the ESF. One of the profiles taken across the edge of the ribbon is highlighted in the inset by a dotted line.

To determine the lateral resolution of the scanner, a highly absorbing polymer ribbon was imaged. The imaged area was 0.5 × 1 mm^2^, scanned with step increments of 1 µm. The excitation pulse energy was 100 nJ, corresponding to a fluence at the focus of 200 mJ/cm^2^, and the detected signals were averaged 4 times. The photoacoustic image (Fig. 3

) is a maximum intensity projection (MIP) formed by extracting the peak-to-peak amplitude of each detected photoacoustic waveform and plotting it as a function of x-y position. Profiles taken across the edge of the ribbon (one of these profiles is highlighted in the inset by a dotted line) were summed up to obtain a single edge spread function (ESF), which exhibits less noise than a single profile. A line spread function (LSF) was then obtained by taking the derivative of the ESF and fitting a Gaussian curve to the data. The lateral resolution was then determined to be 8 µm by measuring the FWHM of the fitted curve (Fig. 3). The axial resolution of the system was quantified by measuring the full width half maximum (FWHM) of the positive peak of the time domain signal shown in Fig. 2(a) and mapping it to distance using the speed of sound (c = 1485 m/s). The axial resolution was estimated to be 21 µm. By contrast, the axial resolution achieved with the fibre sensor with a plane-cleaved tip described in reference [14] was 36 µm. The improved resolution is a consequence of the broader bandwidth and smoother frequency response of the rounded-tip sensor.

**Fig. 4:**
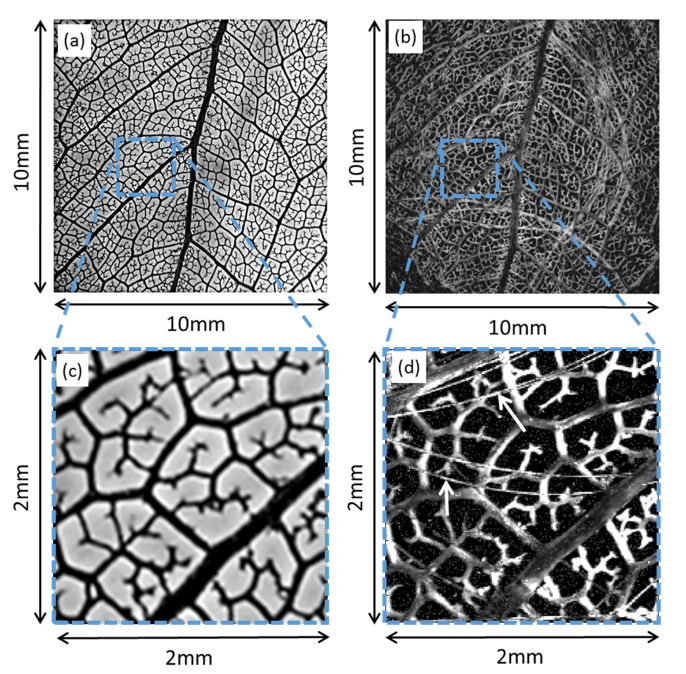
Photoacoustic images of leaf phantom. (a) and (b) show a photograph and a photoacoustic image of the leaf phantom respectively. The scan area was 10 mm × 10 mm with a 10 μm step-size (total of 10^6^ samples). The parameters of the excitation source were λ = 578nm, with a 1.2 ns pulse-width, pulse repetition frequency (PRF) of 3 kHz and <200 nJ pulse energy. The FWHM of the focal spot was 8 μm. The fiber sensor was positioned at a vertical distance of 1.6 mm above the phantom. (c) and (d) show respectively a photograph and a photoacoustic image of a smaller area of the leaf (2 × 2 mm^2^). These areas are indicated by a dotted box in their main respective images. Arrows are used to highlight the presence of some of the carbon fibres.

To further illustrate the imaging performance of the system, a phantom composed of a leaf skeleton, dyed black, and a number of 7 µm diameter carbon fibres were imaged over a 10 × 10 mm^2^ area in steps of 10 µm. Figure 4(a)

shows a photograph of the leaf phantom, without the carbon fibres in place, as it was not possible to take the photograph without disturbing the distribution of the carbon fibres. The sensor was placed 1.6 mm above the phantom at the centre of the imaged area. The pulse energy was 200 nJ, corresponding to a fluence at the focus of 400 mJ/cm^2^, and the detected signals were averaged 4 times. The acquisition time was 22 minutes for a PRF of 3 kHz. Figure 4(b) shows the acquired photoacoustic image. Figure 4(c) and 4(d) show, respectively, an enlarged photograph and photoacoustic image of a smaller part of the leaf phantom (2 × 2 mm^2^) corresponding to the dashed boxes in Figs. 4(a) and 4(b). The photoacoustic images are an accurate representation of the leaf structure shown in the photograph, with the carbon fibres also visible. The large FOV provides a further illustration of the near omnidirectional response of the sensor; for example, at the extremities of the scan area, the sensor is detecting photoacoustic waves at an incident angle of 77 degrees.

## 4. *In-vivo* imaging of mouse ear microvasculature

**Fig. 5:**
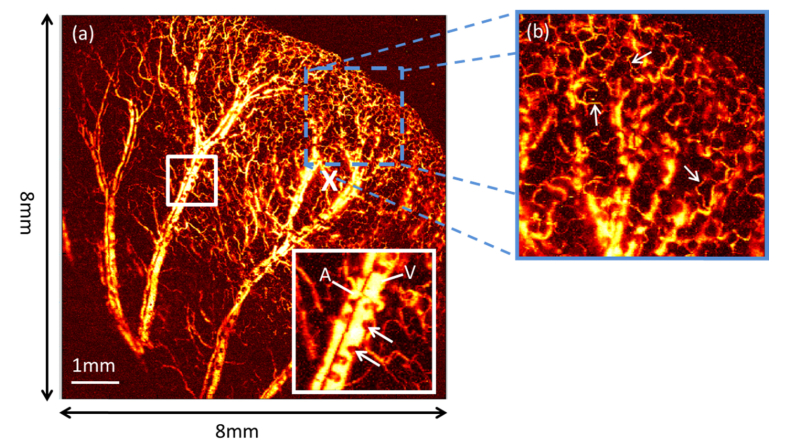
*In vivo* photoacoustic image of mouse ear microvasculature. (a) MIP Image acquired over a scan area of 8 mm × 8 mm with a 10 μm step-size (total of 640 × 10^3^ samples) and an excitation wavelength of 532 nm. A 1.2 ns pulse-width and pulse repetition frequency (PRF) of 5 kHz was used. The FWHM of the focal spot was 8 μm. The position of the fibre sensor is indicated by a white cross and was positioned at a vertical distance of 1.5 mm above the sample. The inset represents a magnified region of interested (solid square box 1.5 × 1.5 mm^2^) highlighting the shadowing caused by the presence of Sebaceous glands, indicated by arrows. (b) Photoacoustic image of a smaller region of interest delineated by a square dotted box in (a) (2 × 2 mm^2^ in steps of 10 µm). The arrows indicate the presence of single capillaries.

The ear of a CD-1 mouse was imaged in order to demonstrate the *in-vivo* capability of the imaging system. Before imaging, the fur on the ear was removed using a commercial hair removal product. The mouse was anaesthetized using a mixture of isofluorance and oxygen at a concentration of 4% for induction, and 1% to 2% for maintenance, with flow rates of 1 l/min. The imaged area was 8 mm by 8 mm with step increments of 10 µm, and the detected photoacoustic signals were averaged 4 times. The fibre sensor was placed 1.5 mm above the sample. The sensor position was offset from the centre of the imaged area, as the positioning of the mouse did not allow placing the sensor directly above the centre of the imaged area. The pulse energy at the tissue surface was between 600 nJ and 800 nJ depending on wavelength. Due to the low NA of the scan lens, this corresponded to a fluence at the tissue surface in the range of 75 to 100 mJ/cm^2^ compared to the ANSI limit of 20 mJ/cm^2^. At the focus, where the irradiance is highest however, the fluence was estimated to be in the range 1.2-1.6 J/cm^2^, which is comparable to that used in other OR-PAM *in vivo* imaging studies [3,4,18,19]. Initially, the laser was operated at a wavelength of 532 nm and a pulse repetition frequency of 5 kHz. The total acquisition time was 9 minutes, limited by the PRF of the laser. Figure 5(a)

shows the acquired photoacoustic image which is an MIP formed as described in section 3. The position of the sensor is indicated by a white cross in Fig. 5(a). Approximately 5 orders of branching can be observed where the vessels sizes range from 10 µm (limited by the scan step size) to 130 µm. Several artery and vein pairs, with diameters of 60 µm and 130 µm respectively (one such artery and vein pair is labelled respectively A and V the inset) can be identified, before branching down to arterioles and venules and then to single capillaries. Shadows in the photoacoustic image caused by presence of microscopic Sebaceous glands can also been seen. These shadows are indicated by arrows in the inset (corresponding to the area indicated by the solid square box in the main figure) of Fig. 5(a). The shadowing is thought to be caused by the higher optical attenuation of Sebaceous glands compared to that of the dermis, therefore reducing the fluence reaching the deeper lying blood vessels. Figure 5(b) highlights a smaller region of interest delineated by a square dotted box in (a), where a few capillaries have been highlighted by arrows.

**Fig. 6:**
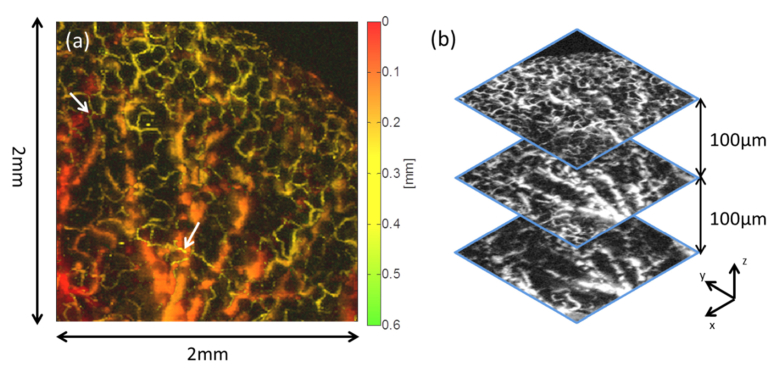
Photoacoustic image of a smaller region of interest delineated by a square dotted box in Fig. 5 (2 × 2 mm^2^ in steps of 10 µm) illustrating depth resolved nature of the image. (a) MIP colour coded for depth. The arrow highlights the presence of a capillary (coloured red) overlaying a deeper laying vessel (coloured in yellow). (b) Slices through the 3D data set for three different depths with a depth separation of 100 µm. A fly-through movie showing successive individual x-y slices through the entire data set can be viewed online (Visualization 1).

Depth information can also be obtained from the time-of-flight of the photoacoustic waves. To produce a 3D depth-resolved image, the envelopes of the detected photoacoustic waveforms were taken and time to depth was mapped via the speed of sound. Figure 6(a)

shows an MIP of this data set, colour coded for depth for the region shown in Fig. 5(b). It illustrates the depth dependent nature of the data; some of the capillaries appearing in yellow (highlighted by arrows) are overlaying deeper vessels coloured in red/orange. The depth separation between the deepest lying feature and the shallowest is approximately 480 µm. Figure 6(b) shows x-y slices through the 3D data set obtained at three different depths each separated by 100 µm. The deeper bottom and middle slices tend to contain the large vessels whereas the small vessels and capillaries are located in the top slice, highlighting the depth resolved nature of the technique. A fly-through movie showing successive individual x-y slices through the entire data set can also be viewed online (Visualization 1).

**Fig. 7:**
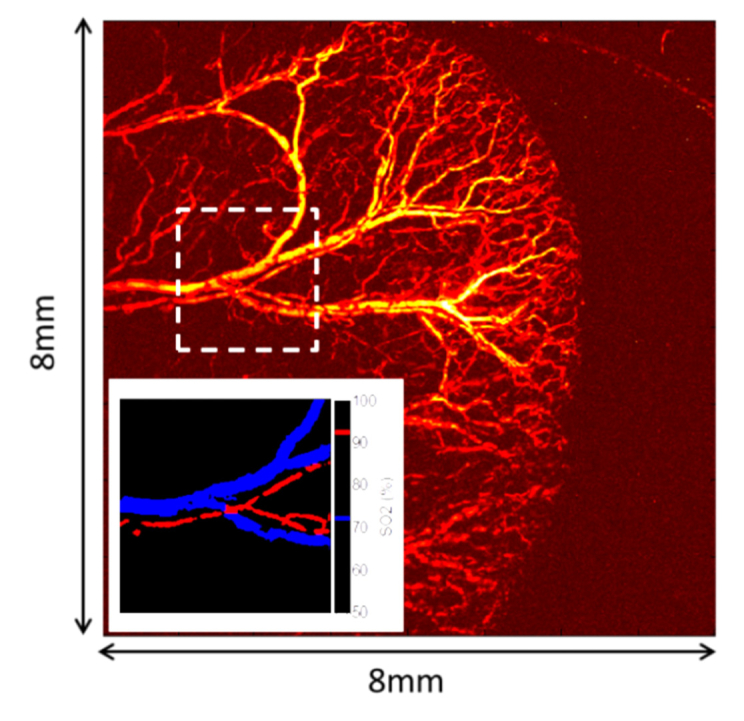
*in-vivo* photoacoustic image of a mouse ear obtained at 578 nm (imaged area: 8 × 8 mm^2^ in steps of 10 µm). The fibre sensor was positioned above the centre of the imaged area at a vertical distance of 2.2 mm above the sample. The inserts shows a map of oxygen saturation, calculated using the mean values of the pixels within each segmented vessel. The region of interest (2 × 2 mm^2^ in steps of 10 µm) is indicated by dotted boxes in the main image.

To demonstrate the ability to obtain an image of blood oxygen saturation, photoacoustic images were acquired at two different wavelengths (571 nm and 578 nm); the photoacoustic image obtained at 578 nm is shown in Fig. 7

. Two blood vessels present in the photoacoustic images (see the dashed box in Fig. 7) were manually segmented and the mean of the pixels within the segmented vessels were calculated for both wavelengths. These values were then used to calculate the vessels’ oxygen saturation using a linear inversion based on Eq. (25) and 26 in [20]. The oxygen saturation within these vessels was calculated to be 72% and 92% for a vein and an artery respectively. The inset of Fig. 7 shows the arteries and vein coloured red and blue respectively.

## 5. Conclusion

In summary, a LS OR-PAM system based on a stationary fibre optic sensor that obviates the need for translational mechanical scanning has been demonstrated. The wide acceptance angle of the sensor allows for larger areas (>1 cm^2^) to be imaged than previously demonstrated with LS OR-PAM systems. The system was demonstrated by acquiring high resolution images of the microvasculature of the mouse ear with sufficient sensitivity and resolution to visualise single capillaries. The broader bandwidth and smoother frequency response of the rounded-tip sensor used in the current study provided an enhanced axial resolution of 21 µm compared to 36 µm achieved with plane-cleaved fibre sensors used in previous work [14]. This enabled a 3D image with sufficient axial resolution to discriminate between vessels lying at different depths to be reconstructed as illustrated in Fig. 6.

The key advantage of LS OR-PAM over conventional OR-PAM implementations is that, by eliminating the need for translational mechanical scanning, it offers the prospect of fast acquisition limited only by the PRF of the excitation laser, the range-ambiguity condition and the galvanometer speed. In the current study, the *in vivo* image acquisition time of 9 minutes was limited by the low PRF of the laser (5 kHz). However, by replacing the laser with a source such as a fibre laser that provides much higher PRF, it should be possible to reduce acquisition time by several orders of magnitude. The maximum PRF which can be used is ultimately limited by the range ambiguity condition, which requires that the time separation between two consecutive excitation pulses is sufficiently long so that the photoacoustic signal generated by the first pulse has time to leave the excited tissue region before the second excitation pulse is delivered. For example, a laser with a 2 MHz PRF would be able to image up to a depth of 742.5 µm (speed of sound of 1485 m/s), which is suitable for most OR PAM applications, while fulfilling the range ambiguity condition. This would allow imaging an area as large as 10 × 10 mm^2^ with a 5 µm step size (corresponding to 4 x 10^6^ A-lines) in just 2 seconds, offering a significant reduction in the acquisition time of existing methods. For smaller FOVs, real time or video rate 3D imaging should be possible for visualising dynamic physiological events.

The small size of the sensor and its large acceptance angle allows it to be placed in close proximity to the target. For example, for skin or eye imaging the sensor could be placed on the surface of the skin or the eyelid, adjacent to the scan area in backward mode and coupled with a drop of ultrasound gel. This configuration is potentially easier to use than conventional piezoelectric based LS OR-PAM systems. To provide a large FOV, these systems are relatively bulky due to the need to locate the ultrasound receiver several cm from the sample to avoid exceeding its small acoustic acceptance angle. Source-detector distances on this scale also typically require for some form of a water bath to provide acoustic coupling thus incurring additional complexity.

In addition, the small size of these fibre optic sensors (<100 µm) suggests they are well suited for endoscopic use. Placing them at the end of a scanning endoscope could find a wide range of applications, such as inspecting the interior of hollow organ systems such as the upper GI tract for the assessment of Barrett oesophagus and other conditions.

## References

[1] Yao J. Wang L. V., “Photoacoustic microscopy,” Laser Photonics Rev. 7(5), 758–778 (2013). 10.1002/lpor.201200060 24416085 PMC3887369

[2] Maslov K. Zhang H. F. Hu S. Wang L. V., “Optical-resolution photoacoustic microscopy for in vivo imaging of single capillaries,” Opt. Lett. 33(9), 929–931 (2008). 10.1364/OL.33.000929 18451942

[3] Hu S. Maslov K. Wang L. V., “Second-generation optical-resolution photoacoustic microscopy with improved sensitivity and speed,” Opt. Lett. 36(7), 1134–1136 (2011). 10.1364/OL.36.001134 21479007 PMC3076123

[4] Yao J. Huang C.-H. Wang L. Yang J.-M. Gao L. Maslov K. I. Zou J. Wang L. V., “Wide-field fast-scanning photoacoustic microscopy based on a water-immersible MEMS scanning mirror,” J. Biomed. Opt. 17(8), 080505 (2012). 10.1117/1.JBO.17.8.080505 23224156 PMC3418511

[5] Kim J. Y. Lee C. Park K. Lim G. Kim C., “Fast optical-resolution photoacoustic microscopy using a 2-axis water-proofing MEMS scanner,” Sci. Rep. 5(1), 7932 (2015). 10.1038/srep07932 25604654 PMC4300456

[6] Xie Z. Jiao S. Zhang H. F. Puliafito C. A., “Laser-scanning optical-resolution photoacoustic microscopy,” Opt. Lett. 34(12), 1771–1773 (2009). 10.1364/OL.34.001771 19529698

[7] Xie Z. Chen S.-L. Ling T. Guo L. J. Carson P. L. Wang X., “Pure optical photoacoustic microscopy,” Opt. Express 19(10), 9027–9034 (2011). 10.1364/OE.19.009027 21643156 PMC3324262

[8] Yuan Y. Yang S. Xing D., “Optical-resolution photoacoustic microscopy based on two-dimensional scanning galvanometer,” Appl. Phys. Lett. 100(2), 023702 (2012). 10.1063/1.3675907

[9] Song W. Wei Q. Feng L. Sarthy V. Jiao S. Liu X. Zhang H. F., “Multimodal photoacoustic ophthalmoscopy in mouse,” J. Biophotonics 6(6-7), 505–512 (2013). 10.1002/jbio.201200061 22649053 PMC3986594

[10] Kang H. Lee S.-W. Lee E.-S. Kim S.-H. Lee T. G., “Real-time GPU-accelerated processing and volumetric display for wide-field laser-scanning optical-resolution photoacoustic microscopy,” Biomed. Opt. Express 6(12), 4650–4660 (2015). 10.1364/BOE.6.004650 26713184 PMC4679244

[11] Shi W. Hajireza P. Shao P. Forbrich A. Zemp R. J., “In vivo near-realtime volumetric optical-resolution photoacoustic microscopy using a high-repetition-rate nanosecond fiber-laser,” Opt. Express 19(18), 17143–17150 (2011). 10.1364/OE.19.017143 21935076

[12] Li L. Yeh C. Hu S. Wang L. Soetikno B. T. Chen R. Zhou Q. Shung K. K. Maslov K. I. Wang L. V., “Fully motorized optical-resolution photoacoustic microscopy,” Opt. Lett. 39(7), 2117–2120 (2014). 10.1364/OL.39.002117 24686689 PMC4048805

[13] Allen T. J. Zhang E. Beard P. C., “Large-field-of-view laser-scanning OR-PAM using a fibre optic sensor,” in Proc. of SPIE, Photons Plus Ultrasound: Imaging and Sensing, Oraevsky A. A. Wang L. V., eds. (2015), Vol. 9323, p. 93230Z.

[14] Guggenheim J. A. Li J. Allen T. J. Colchester R. J. Noimark S. Ogunlade O. Parkin I. P. Papakonstantinou I. Desjardins A. E. Zhang E. Z. Beard P. C., “Ultrasensitive plano-concave optical microresonators for ultrasound sensing,” Nat. Photonics 11(11), 714–719 (2017). 10.1038/s41566-017-0027-x

[15] Zhang E. Z. Beard P. C., “Characteristics of optimized fibre-optic ultrasound receivers for minimally invasive photoacoustic detection,” in Proc. of SPIE, Photons Plus Ultrasound: Imaging and Sensing Oraevsky A. A. Wang L. V., (2015), 9323, pp. 932311.

[16] Zhang E. Laufer J. Beard P., “Backward-mode multiwavelength photoacoustic scanner using a planar Fabry-Perot polymer film ultrasound sensor for high-resolution three-dimensional imaging of biological tissues,” Appl. Opt. 47(4), 561–577 (2008). 10.1364/AO.47.000561 18239717

[17] Guggenheim J. A. Zhang E. Z. Beard P. C., “A Method for Measuring the Directional Response of Ultrasound Receivers in the Range 0.3-80 MHz Using a Laser-Generated Ultrasound Source,” IEEE Trans. Ultrason. Ferroelectr. Freq. Control 64(12), 1857–1863 (2017). 10.1109/TUFFC.2017.2758173 28976314

[18] Wissmeyer G. Soliman D. Shnaiderman R. Rosenthal A. Ntziachristos V., “All-optical optoacoustic microscope based on wideband pulse interferometry,” Opt. Lett. 41(9), 1953–1956 (2016). 10.1364/OL.41.001953 27128047

[19] Dong B. Hao L. Zhang Z. Zhang K. Chen S. Sun C. Zhang H. F., “Isometric multimodal photoacoustic microscopy based on optically transparent micro-ring ultrasonic detection,” Optica 2(2), 169–176 (2015). 10.1364/OPTICA.2.000169 PMC5969522 29805988

[20] Cox B. Laufer J. G. Arridge S. R. Beard P. C., “Quantitative spectroscopic photoacoustic imaging: a review,” J. Biomed. Opt. 17(6), 061202 (2012). 10.1117/1.JBO.17.6.061202 22734732

